# Treatment of an Epithelial Cancer of the Tongue with Injections of Nitrate of Silver

**Published:** 1877-05

**Authors:** W. H. Vittum

**Affiliations:** Baraboo, Wis.


### TREATMENT OF AN EPITHELIAL CANCER OF THE TONGUE WITH INJECTIONS OF NITRATE OF SILVER.—RECOVERY.

By. DR. W. H. VITTUM, M. D., of Baraboo, Wis.

The treatment of malignant tumors has always been a *bete noire* to the profession, and any advance in the direction of a successful struggle with them will be hailed with joy. The object of the present paper is to give an account of a case of epithelioma of the tongue, successfully treated by parenchymatous injections of nitrate of silver.

On the 26th of Dec. 1876, Mr. M. N., aet 59, applied to the writer and his partner, Dr. M. M. Davis, for treatment of a "bunch in his tongue." Upon examination, a tumor of the size of an English walnut was discovered, about one inch back of the tip of the tongue, and situated on the right side. The entire thickness of the tongue was involved. The surface of the tumor was covered with roughened and warty excrescences, and all the tissues in the immediate neighborhood of the growth were in an infiltrated condition. The tumor had increased in bulk very rapidly, having attained its present size in about two months. There was a great deal of lancinating pain, accompanied by extreme tenderness and a swollen condition of the whole organ.

The tenderness was so great that eating solids was impossible. The neighboring lymphatic glands were intact. The diagnosis of epithelial cancer of the tongue was made.

Having seen an account of the successful treatment of epithelioma with injections of nitrate of silver by Dr. Wilde, in the Chicago Medical Journal and Examiner, for March, 1876, we determined to try their efficacy in this case.

The tumor was accordingly injected in several places with a solution of nitrate of silver, (one grain to the ounce). The intention was, as Wilde suggested, to inject enough to saturate the tumor. This saturation was repeated daily for seven days, and then every alternate day until ten sittings had been held, when the tumor was pronounced cured. There was a very marked amelioration of symptoms as early as the day after the second injection.

Such prompt and complete success with a tumor which was so rapidly growing, and whose malignant nature was so evident, should fill us with the hope that at least one species of cancer (when it occurs in accessible situations) may be stricken from the long list of incurable diseases.

